# Real-World Evaluation of AI-Driven Diabetic Retinopathy Screening in Public Health Settings: Validation and Implementation Study

**DOI:** 10.2196/67529

**Published:** 2025-09-09

**Authors:** Mona Duggal, Anshul Chauhan, Vishali Gupta, Ankita Kankaria, Deepmala Budhija, Priyanka Verma, Vaibhav Miglani, Preeti Syal, Gagandeep Kaur, Lakshay Kumar, Naveen Mutyala, Rishabh Bezbaruah, Nayanshi Sood, Ashleigh Kernohan, Geeta Menon, Luke Vale

**Affiliations:** 1 ICMR-National Institute for Research in Digital Health and Data Science (NIRDHDS), Ansari Nagar East, New Delhi, 110029, India, 91-11-26588803; 2 Advanced Eye Centre, Post Graduate Institute of Medical Education and Research, Chandigarh, 160012, India; 3 Department of Community Medicine and School of Public Health, Post Graduate Institute of Medical Education and Research, Chandigarh, 160012, India; 4 National Institute of Transforming India (NITI) Aayog, Delhi, India; 5 Department of Ophthalmology, Department of Health and Family Welfare, Mohali, Punjab, India; 6 Global Health Economics Centre, Public Health and Policy, London School of Hygiene and Tropical Medicine, London, United Kingdom; 7 Frimley Health NHS Foundation Trust, Frimley, United Kingdom

**Keywords:** diabetic retinopathy, artificial intelligence, validation, implementation, integration, Screening, public health settings, screening

## Abstract

**Background:**

Artificial intelligence (AI) algorithms offer an effective solution to alleviate the burden of diabetic retinopathy (DR) screening in public health settings. However, there are challenges in translating diagnostic performance and its application when deployed in real-world conditions.

**Objective:**

This study aimed to assess the technical feasibility of integration and diagnostic performance of validated DR screening (DRS) AI algorithms in real-world outpatient public health settings.

**Methods:**

Prior to integrating an AI algorithm for DR screening, the study involved several steps: (1) Five AI companies, including four from India and one international company, were invited to evaluate their diagnostic performance using low-cost nonmydriatic fundus cameras in public health settings; (2) The AI algorithms were prospectively validated on fundus images from 250 people with diabetes mellitus, captured by a trained optometrist in public health settings in Chandigarh Tricity in North India. The performance evaluation used diagnostic metrics, including sensitivity, specificity, and accuracy, compared to human grader assessments; (3) The AI algorithm with better diagnostic performance was integrated into a low-cost screening camera deployed at a community health center (CHC) in the Moga district of Punjab, India. For AI algorithm analysis, a trained health system optometrist captured nonmydriatic images of 343 patients.

**Results:**

Three web-based AI screening companies agreed to participate, while one declined and one chose to withdraw due to low specificity identified during the interim analysis. The three AI algorithms demonstrated variable diagnostic performance, with sensitivity (60%-80%) and specificity (14%-96%). Upon integration, the better-performing algorithm AI-3 (sensitivity: 68%, specificity: 96, and accuracy: 88·43%) demonstrated high sensitivity of image gradability (99.5%), DR detection (99.6%), and referral DR (79%) at the CHC.

**Conclusions:**

This study highlights the importance of systematic AI validation for responsible clinical integration, demonstrating the potential of DRS to improve health care access in resource-limited public health settings.

## Introduction

The global prevalence of diabetes is growing [1], leading to increased vision loss and blindness associated with it [2]. There is an urgent need for diabetic retinopathy (DR) screening programs to identify vision-threatening DR to enable timely treatment [3,4]. However, this rising prevalence is straining health care systems already struggling to improve care and manage health care costs [4].

Despite its critical role, DR screening (DRS) remains limited in many low-resource settings. Conventional screening with trained human graders is often costly, time-consuming, and challenging to scale [5,6]. The gap between eye care needs and ophthalmologist availability exacerbates public health challenges [7]. In India, DR management faces challenges due to limited screening programs, low public awareness, and poor understanding of routine retinal exams [8]. Artificial intelligence-driven DRS enables faster, more affordable, and efficient screening, especially in underserved areas [9,10]. It allows noneye health professionals to conduct screenings and refer without specialists [11].

AI algorithms have demonstrated performance comparable to or exceeding human experts in DR classification [12,13]. However, concerns exist about their suboptimal performance in real-world settings and across diverse populations [14]. Real-world validation is critical to ensure AI algorithms perform accurately in diverse settings, as disease prevalence, image quality, and patient-related factors may differ from the training dataset [15-17]. Prospective studies are essential for evaluating AI systems in the contexts where they will be deployed [18]. Hence, integrating AI into clinical practice requires alignment with clinical workflows and stronger evidence on its real-world accuracy and user experiences [11,14].

This is the first Indian study to validate multiple commercial AI algorithms for DRS and to assess the feasibility of implementing a validated AI system in public health settings.

## Methods

### Study Design

**Figure 1.**
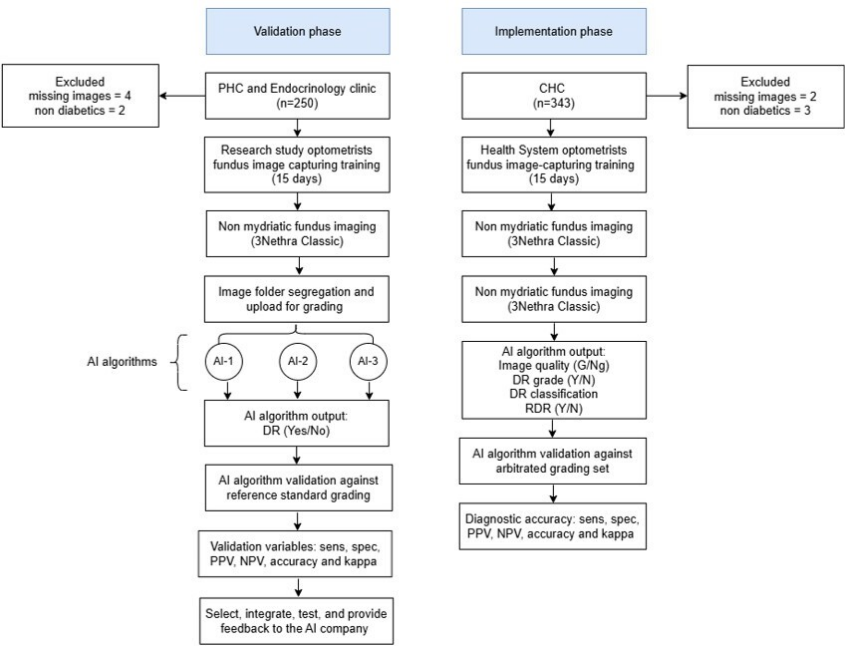
Study flow chart. Implementation phase occurred after the completion of the validation phase (ie, not simultaneously). AI: artificial intelligence; CHC: community health center; DR: diabetic retinopathy; PHC: primary health center; PPV: positive predictive value; NPV: negative predictive value; RDR: referable diabetic retinopathy.

The study prospectively validated three DR detection AI algorithms (validation phase) and assessed the technical feasibility of implementing a validated AI algorithm in public health settings (implementation phase). The STARD (Standards for Reporting of Diagnostic Accuracy Studies) checklist [19] was used to report the completeness and transparency of the diagnostic accuracy study and the iCHECK-DH framework to enhance the completeness and transparency of reporting related to the digital health implementation components [20]. Figure 1 summarizes the study design.

### Ethical Considerations

The study received approval from the Postgraduate Institute of Medical Education and Research (PGIMER) Institutional Ethics Committee (PGI/IEC/2020/001342) and followed the recommendations of the Declaration of Helsinki. The study was prospectively registered with the Clinical Trials Registry India (CTRI/2022/10/046185). Individuals aged > 30 years with a history of diabetes mellitus were screened for DR, following the National Program for Prevention and Control of Non-Communicable Diseases [21]. Informed consent was obtained, and their routine care remained unchanged. All data used in this study were anonymized prior to analysis, with no personal identifiers retained.

### Study Site (Real-World Settings)

Validation was conducted from March to June 2021 at the Department of Endocrinology, PGIMER, Chandigarh, and a Primary Health Center (PHC) in Khizrabad, District Mohali, Punjab. The AI-enabled DRS was implemented between February 2022 and June 2022 at a community health center (CHC) in Badhani Kalan, Moga District, Punjab, India.

### Sample Size

Sample sizes (validation=256; implementation=348) were calculated assuming DR prevalence of 17%, nongradable image rates of 18.4% in validation and 30.3% during implementation, 70% sensitivity, 86% specificity, 95% CI, and nonresponse rates of 10% and 15% in validation and implementation, respectively [3,22-25].

### AI Algorithms

Based on a scoping review, five AI companies that used cloud-based AI (four Indian, one international) were invited. Before validation, they received the study objectives and camera or image specifications.

### Fundus Image Acquisition

#### Training of Optometrists

Two experienced optometrists were trained for 15 days at the Advanced Eye Centre (AEC) and PHC settings before validation (Table S1 in Multimedia Appendix 1) [26]; two distinct optometrists received similar training for implementation in Moga district. Training covered identifying good-quality images [27] and capturing additional images when needed. They were supervised until proficient in independent data collection and imaging.

#### Camera

All participants underwent nonmydriatic, two-field (macula and disc-centered), 45° FOV fundus photography using a 3Netra Classic Portable Benchtop Fundus Camera (Forus Health) [26].

#### Recruitment Process

During validation, participants were recruited through Accredited Social Health Activist (ASHA) workers at PHCs and by research staff in the endocrinology department . The study did not alter patients’ routine care during the recruitment process. During implementation, recruitment at the CHC was assisted by a nurse from the medicine clinic.

#### Darkroom

[To minimize ungradable images from nonmydriatic cameras that are a key challenge for real-world DRS programs, [28]dark rooms with sealed windows and ventilators were set up at PHC Khijrabad and at CHC Badhani Kalan (Figure S1 in Multimedia Appendix 1), with participants seated in the dark for ≥2 minutes to achieve physiological mydriasis before imaging.

#### Image Grading

Image identifiers were removed, and images remained unprocessed before AI analysis. The research optometrist sorted and uploaded them by eye laterality (ie, right and left eye). AI companies had no direct access; only the uploading optometrist handled the images per the agreement with the companies. A separate, role-based account was created for image upload and grading, accessible only to the optometrist. Audit logs tracked access, and secure file transfer protocols protected the transmission of images and the human grader had similar restricted access. Validation images were not used for training or testing the AI.

#### AI Grading Protocol

The AI algorithms’ screening outcomes were inconsistent in providing DR stages (mild nonproliferative diabetic retinopathy (NPDR)), moderate NPDR, severe NPDR, and proliferative diabetic retinopathy (PDR)), diabetic macular edeme (DME) (yes/no), and referable DR (RDR) (Figure S2 in Multimedia Appendix 1). Hence, the DR grade (yes/no) was chosen to validate the AI algorithms discussed elsewhere [29].

#### Reference Standard Grading

All AI screening outputs were manually graded by human graders using the International Classification for Diabetic Retinopathy (ICDR) classification system [30]. Images were labeled as “gradable” or “non-gradable,” and DR as “present” or “absent.” DR was classified as mild, moderate, or severe NPDR and PDR [30]. Microaneurysms that are early signs of DR, indicate mild NPDR [31]. Any DR mentioned above, including moderate NPDR were considered referable DR (RDR) [30]. DME was defined as hard exudates with or without foveal involvement [3]. Images with ≥80% visibility and clear view up to the third vascular branch were considered gradable and assessed for DR [27].

- Validation phase*:* Two masked human graders (HG1 and HG2), a trained optometrist with three years of grading experience, and a retina-trained ophthalmologist with seven years of grading experience, with different institutional affiliations, independently graded all the fundus images. A senior vitreoretinal expert with 25 years of grading experience re-evaluated 224 images from 56 participants (75%) where HG1 and HG2 disagreed on DR presence. A strong level of agreement [32] (κ=0.85) was observed between HG2 and the senior retina specialist; hence, HG2 grading was considered the reference standard (RS) for AI validation.
- Implementation phase*:* All images were independently graded by two masked human graders (HG1 and HG2), who were different from those involved in the validation phase. In cases of disagreement, the grades were reviewed and adjudicated by a senior vitreoretinal specialist with over eight years of experience in grading. The final consensus-based, arbitrated dataset served as the RS and was used for all statistical analyses.

#### AI Integration and Implementation

The better-performing AI algorithm was integrated into the 3Netra Classic and pilot-tested for two weeks at AEC and District Hospital (DH), Mohali. This phase ensured hardware-software compatibility, assessed internet connectivity, and included dummy tests to validate outputs before implementation. The two-week testing at PGIMER and DH Mohali validated the full AI workflow, identified technical issues, assessed reliability in clinical settings, and highlighted data and reporting bottlenecks.

This feedback led to key refinements, including better internet connectivity, mandatory clinical variables, faster result turnaround, and local data storage on the National Institute of Transforming India (NITI) server for compliance. Final adjustments were implemented at Moga and Mohali sites (Figure S3 in Multimedia Appendix 1). The AI algorithm did not undergo additional training during the implementation period. Testing was conducted by optometrists, supported by a research optometrist, and supervised by the data scientist and principal investigator. The research team coordinated with the AI company to address feedback, and the machine learning scientist assisted with preimplementation adjustments.

Poststudy, a follow-up mechanism was established to ensure continued service delivery (6 mo), with the optometrist monitored for adherence to screening protocols. Notably, the retinal camera and adjustable stand were retained at the CHC rather than being reclaimed as study assets, reinforcing sustainability through local ownership and continuity of service.

#### Referral Recommendation and Telephonic Follow-Up

Referral were based on cases diagnosed as moderate or more severe, as well as those with DME [30]. Participants requiring follow-up were contacted by phone one month after their DRS appointment to assess compliance, with up to three contact attempts made to gather this information.

### Data Analysis

The study data was collected using Research Electronic Data Capture (REDCap) [33]. Deidentified data were downloaded from REDCap and imported into Stata/IC (version 15.1; StataCorp) [34] for analysis.

The AI platform’s sensitivity, specificity, positive predictive value (PPV), and negative predictive value (NPV) were estimated with 95% exact binomial CIs. A *P* value of <·05 was considered significant for all statistical tests. A κ value was calculated monthly during the implementation phase to assess the optometrists learning in image acquisition and the AI algorithm’s performance in image quality and DR diagnosis. This measured the agreement between AI and RSs for image quality and DR diagnosis. The folders with missing bilateral or macula-centered images and people without diabetes mellitus were excluded from the analysis. All analyses were conducted on an eye-wise basis.

## Results (Implementation)

Three AI algorithm companies Leben Care Health Services, Retinal AI Diagnostic Software, and SigTuple Technologies. Each AI platform was masked from the others and randomly assigned labels such as AI 1, AI 2, and AI 3. One Indian AI algorithm declined participation, and an Food and Drug Administration (FDA)-approved algorithm was excluded due to low specificity in interim analysis. Two Indian AI companies approached during the implementation phase but were not included.

### Sociodemographic Details of the Study Participants

Among the 250 participants in the validation phase, 182 were recruited at the PHC Khijrabad and 68 from the endocrinology clinic. The mean age of the participants was 53.5 (SD 7) years in the PHC and 47.2 (SD 4) years in the Endocrinology clinic, respectively. Overall, 87/250 (48.3%) participants in the PHC group were men and 95 (51.7%) were women, and those in the Endocrinology group included 38 (55.8%) men and 30 (44.1%) women. In the implementation phase, 343 participants were recruited. The participants’ mean age was 58.48 (SD 10.41) years, with 202/343 (59%) women and 140 (41%) men (Table 1).

**Table 1.** Study participants demographic characteristics.

Characteristics	Validation phase (n=250)	Implementation phase (n=343)
Gender n (%)		
Men	121(48.4%)	141 (41)
Women	129(51.6%)	202 (59)
Age (years), mean (SD)	54.4 (13.8)	58.4 (10.4)
Duration of diabetes (years), mean (SD)	7.1 (6.1)	6.2 (5.9)
Education, n (%)		
Illiterate	72(28.8)	135(39.4)
Primary	79(31.6)	125(36.4)
Matriculation	70(28)	63(18.4)
Secondary	14(6.8)	8(2.3)
Graduation and above	15(6)	12(3.5)

#### Validation Phase

**Figure 2.**
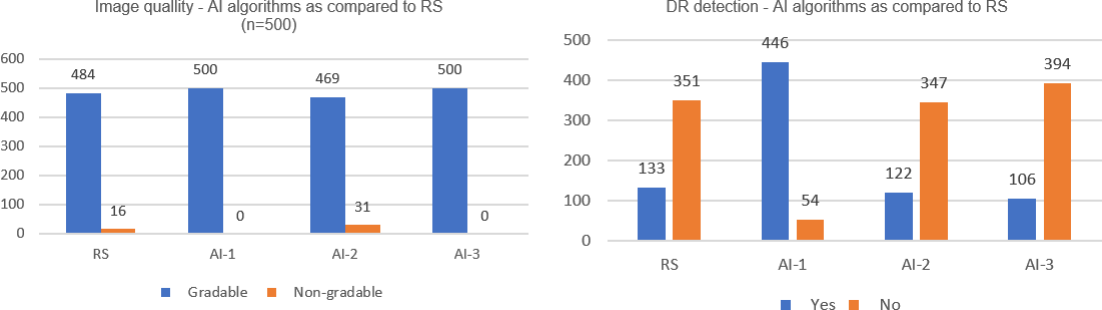
Image quality of artificial intelligence (AI) algorithms as compared to the reference standard (RS). DR: diabetic retinopathy.

The analysis included 1099 fundus images of 500 eyes from 250 participants. According to the RS, 484 (96·8%) of eyes were gradable, with 16 (3·2%) being ungradable. AI 1, AI 2, and AI 3 achieved excellent gradability (100%, 93.8%, and 100%, respectively). The RS detected DR in 133 (27.48%) of the eyes; AI 1 detected notably more cases of DR in 446 (89.2%) eyes compared with AI 2, 122 (26.22%), and AI 3, 106 (21.2%) (Figure 2).

AI 3 showed the best performance with specificity of detecting DR, 96.01% (93·24‐97·72); sensitivity, 68.42% (59·71‐76.05); PPV, 86.67% (78.31‐92.26); accuracy, 88.43%; and agreement with the RS (κ=0·65) and was selected for implementation (Table S2 in Multimedia Appendix 1). The validation results have been presented elsewhere [29].

Based on our study findings and recommendations from the project technical oversight committee which comprised ophthalmologists, public health experts, academicians, technologists, and research scientists providing technical expertise and strategic direction to evaluate, guide, and strengthen the project’s scientific rigor. The AI company was recommended to train its algorithm for DR stages, DME grading, and referral guidelines, incorporating inputs on camera integration, connectivity, and platform functionality. Changes were completed within four months, and implementation began at CHC, with testing images excluded from the final set (Table S3 in Multimedia Appendix 1). Changes were incorporated within four months and implemented at CHC.

#### Implementation Phase

**Figure 3.**
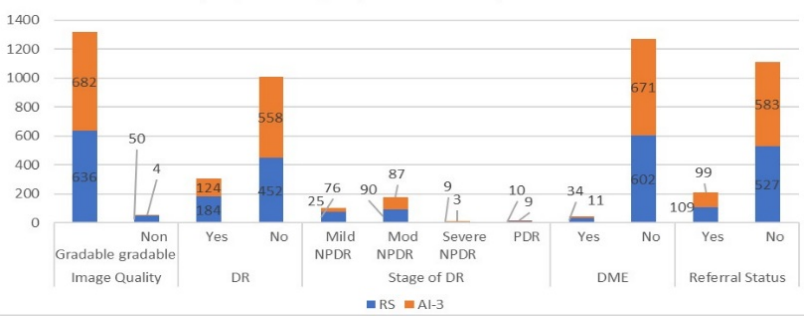
Diabetic retinopathy screening outputs of AI as compared to the reference standard at Community Health Center Moga. AI: artificial intelligence; DME: diabetic macular edeme; DR: diabetic retinopathy; PDR: proliferative diabetic retinopathy; NPDR: nonproliferative diabetic retinopathy.

During the implementation phase, 1372 fundus images were captured from 686 eyes of 343 participants. AI-3 exhibited slightly higher image gradability (682, 99.5%) than the RS (336, 92.71%). AI-3 identified 124 (18.2%) of participants with DR, while the RS detected 189 (28.9%). Moderate NPDR was most common (AI-3 at 87 (12.8%) and RS at 90 (14.1%). DME detection was lower for AI-3, 11 (1.6%) than the RS, 34 (5.3%). Referral rates were almost similar, (AI-3: 99; 14.5%) and RS (109, 17.1%) (Figure 3).

Among the 50/686 (7.3%) ungradable eyes graded by HG, 21 (42%) had cataracts, while none labeled as ungradable by AI had cataract. The sensitivity, specificity, PPV, and NPV of AI-3 algorithm for DR detection were 99.6%, 64.7%, 87.4%, and 98.3%, respectively. For DME, specificity, PPV, and NPV were 99.7%, 81.8%, and 96%, respectively. However, sensitivity is relatively low (26.5%); for detecting RDR, sensitivity and specificity were 78.9% and 98.1%. For image gradability, the AI’s sensitivity was excellent at 100%, but specificity was low (8%) (Table 2).

**Table 2.** Sensitivity, specificity, PPV^a^, NPV^b^ of the AI^c^ algorithm for various outcome variables with a reference standard.

Outcome variables	Image gradability	DR^d^ grade	DME^e^ grade	RDR^f^
Sensitivity (%)	100	99·6	26·5	78·9
Specificity (%)	8	64·7	99·7	98·1
PPV (%)	93·2	87·4	81·8	89·6
NPV (%)	100	98·3	96	95·7
κ value	0.69	0.72	0.38	0.81

a PPV: Positive predictive value.

b NPV: Negative predictive.

c AI: artificial intelligence.

d DR: Diabetic retinopathy.

e DME: Diabetic macular edeme.

f RDR: referable diabetic retinopathy.

### κ Statistic Variation Across the Study Period

**Figure 4.**
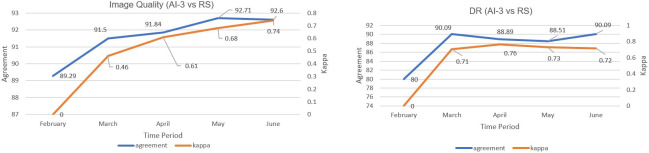
Agreement and kappa statistics for image quality and DR grade between AI and reference standard. AI: artificial intelligence; DR: diabetic retinopathy; RS: reference standard.

Figure 4 shows κ variations for image quality and DR grades over 4.5 months when the health system optometrist captured the images. κ values of image quality increased from 0 in February to 0.74 in June, and for DR grade from 0 to 0.71 in June. This steady improvement could be attributed to the enhanced image quality captured by the optometrist over the study period and higher; in DR, this increase can be linked to better image quality and higher detection, with slight dips due to variation in DR severity or case complexity.

### Participant Referral and Follow-Up

Of 64 referred participants, 28 (43.8%) were contacted; only 9 (14%) adhered to referral advice and visited an ophthalmologist for a review. Of these, 1 received an Optical Coherence Tomography referral, 5 went to facilities without eye care services, 1 received eye drops, 1 was advised antivascular endothelial growth factor injection, 1 left after receiving laser treatment, and 1 had a follow-up visit.

Reasons for nonadherence included harvesting season (n=11), lack of family support (n=2), time constraints (n=2), extended absence from home (n=1), financial dependence on family (n=2), and comorbidities (n=1).

## Discussion

### Principal Findings

This study is among the first in India to validate multiple AI algorithms for DRS, critically assessing their technical feasibility for integration into real-world public health settings. We evaluated the best-performing AI algorithm using images captured by a skilled research optometrist. This study outlines the Government of India’s efforts to foster an ecosystem that ensures the integration of responsible AI technologies before application to end users [35].

In our study, AI system performance during validation varied significantly: sensitivity (59.7%97.74%), specificity (14.25%96.01%), PPV (30.16%86.67%), NPV (85%94.34%), and accuracy (37.19%88.43%). A multicenter study by Lee et al systematically compared seven AI-based DRS algorithms, revealing high NPVs (82.72%93.69%) but widely varying sensitivities (50.98%85.90%) in real-world performance [36]. In pivotal clinical trials for the IDX AI algorithm, the FDA’s benchmark for superiority was set at 85% sensitivity and 82.5% specificity [13]. Although AI-3 sensitivity (68.3%) was below the FDA’s >85% threshold, it outperformed other AIs in other diagnostic metrics: specificity (96.01%), PPV (86.67%), NPV (88.92%), and accuracy (88.43%). Prospective validation of a DRS algorithm at two Indian tertiary eye care hospitals demonstrated 89% sensitivity and 92% specificity on nonmydriatic images [37].

However, there are challenges in comparing algorithms using published results due to the variations in study methodologies [38]. One challenge is the dependence of an AI algorithm’s accuracy on the quality of the retinal images obtained [27]. Our study included ungradable images in validation, contrasting with many studies that preprocess or exclude lower-quality images in their training data sets [12,13,39-41] (Figure S6 in Multimedia Appendix 1). Excluding these images fails to reflect real-world settings, potentially lowering the algorithm’s performance [13,41]. To date, validation studies demonstrate that most AI algorithms using mydriatic fundus images achieve high diagnostic accuracy [42]. In the validation phase, the dark room environment facilitated nonmydriatic conditions, yielding 0% ungradability for AI-1 and AI-3, 6.2% for AI-2, and 3.2% by human grader, contrasting with 1830% in LMIC settings per a systematic review [38,42].

Cataracts are a leading cause of ungradable images [28]. However, no images with cataract or other media opacities were classified as ungradable during implementation, potentially affecting AI-3 specificity (Table 2). Notably, the AI-3 algorithm failed to detect 21 (42%) eyes with cataracts, categorized as gradable. The AI-3 algorithm’s sensitivity increased significantly from 68.4% to 99.6%, while specificity decreased from 96% to 64.7% between the validation and implementation phases. The sensitivity likely improved post validation due to algorithm training (Table S2 in Multimedia Appendix 1). The presence of cataracts, affecting media opacity and gradability, likely could have decreased specificity by increasing false positives [43]. After algorithmic training, AI-3 detected DME (Table 2) and sensitivity (26.5%), highlighting the need for improved training to enhance DME sensitivity and subsequently achieve higher RDR sensitivity (78.9%).

Optometrists serve as frontline eye care providers globally and are ideally positioned for task-sharing in DRS. Their integration into DRS and care pathways is well-established in diverse models worldwide, supporting sustainable and scalable eye care delivery [44-46]. In this study, a health system optometrist was trained and engaged in DRS at the CHC, with oversight from the research team. Over the study period, image quality and DR detection improved, reflecting a positive learning curve (Figure 3). These findings highlight the potential of optometrist-led AI-assisted screening in strengthening task-shifting models and expanding access to DR care in resource-limited settings [47-49]. Implementing an opportunistic AI-enabled DRS model holds promise for enhancing detection rates [47]. However, our study’s adherence to referral recommendations remains suboptimal, with approximately 14% of participants attending recommended follow-up visits at an eye care facility. Key barriers to referral adherence include awareness gaps, logistical challenges (ie, travel, DR related cost), and persisting health system limitations, including weak referral pathways and poor patient tracking [48]. Personalized approaches, such as phone calls, voicemails, and detailed result letters, are shown to be effective in improving referral adherence [49]. Low referral adherence underscores the need for effective, coordinated referral pathways before introducing new screening models, while recognizing the vital role of teleophthalmology in such systems [47,50].

A notable strength of this study is its real-time implementation, conducted during regular clinics by a health system optometrist within a public health care setting. This marks a significant milestone that is likely to substantially enhance DRS services. The study showed that training and monitoring fundus image quality could significantly improve the effectiveness of the AI-enabled DRS program.

However, several limitations should be considered when interpreting the results. Using a single fundus camera may limit generalizability, as AI performance may vary across camera models and imaging conditions. Additionally, the study relied on a specific AI algorithm, which may not account for variations in other models or evolving AI systems. Setting-specific constraints such as workflow integration and infrastructure availability could also impact scalability. Furthermore, potential biases in real-world data, such as variations in patient demographics, image quality, and disease prevalence, may influence AI performance and limit broader applicability. Integrating DRS was feasible; however, we could monitor the optometrist only for three months poststudy. Regular monitoring is crucial for program sustainability and healthcare provider motivation.

### Lessons Learnt

Operational challenges in DRS included limited patient access due to long travel distances, poor transport, and low awareness; inadequate infrastructure; and ergonomic barriers affecting both patients and screeners. Uncontrolled lighting and power issues led to 26% ungradable images. Adaptive measures, such as transport support, ergonomic adjustments, darkroom setups, and equipment reinforcements, raised image gradeability to 95.6% and improved efficiency. Addressing these barriers through infrastructure upgrades, controlled environments, and community facilitation is essential for sustainable DRS in primary health care systems.

In conclusion, this study highlights the essential role of systematic AI validation in integrating technology responsibly into clinical workflows. By demonstrating AI’s feasibility for DRS in Indian public health settings, our findings support scalable solutions to improve health care accessibility in resource-constrained contexts across the Global South. However, long-term sustainability and large-scale implementation will require ongoing funding, a robust workforce, and effective policy integration. Further research is needed to evaluate the large-scale deployment of AI-driven screening strategies, examining their clinical effectiveness, cost-effectiveness, and the challenges of implementing them in real-world settings.

## Supplementary material

10.2196/67529 Multimedia Appendix 1 Supplementary materials.

10.2196/67529 Checklist 1 iCHECK-DH: Guidelines and Checklist for the Reporting on Digital Health Implementations.

## Abbreviations

AI: artificial intelligence
CHC: community health center
DME: diabetic macular edeme
DR: diabetic retinopathy
DRS: diabetic retinopathy screening
NPDR: nonproliferative diabetic retinopathy
NPV: negative predictive value
PDR: proliferative diabetic retinopathy
PHC: primary health center
PPV: positive predictive value
RDR: referable diabetic retinopathy
RS: reference standard
STARD: Standards for Reporting of Diagnostic Accuracy Studies
