# Supplementary material for: Real-World Evaluation of AI-Driven Diabetic Retinopathy Screening in Public Health Settings: Validation and Implementation Study
Source: JMIR Med Inform. 2025 Sep 9;13:e67529. doi: 10.2196/67529 (PMC12419978; doi:10.2196/67529)

**Real-World Evaluation of AI-Driven Diabetic Retinopathy Screening in Public Health Settings: Validation and Implementation Study**

**Supplementary Table 1: Training module content**

|  | **Content** | **Material** | **Day** |
| --- | --- | --- | --- |
| 1 | How to record history (pen and paper) | Video/paper | Day 1 |
| 2 | Patient sitting while image capturing | Video | Day 1 |
| 3 | Patient positioning while image capturing | Video | Day 1 |
| 4 | Patient posture while image-capturing | Video | Day 1 |
| 4.1 | Patient posture while image-capturing | Video | Day 1 |
| 5 | Entering variable on the camera software | Video | Day 1 |
| 6 | Light focus and image capture | Video | Day 2 |
| 7 | Image saving source in laptop | Video | Day 2 |
| 8 | Image containing folders (Right and Left) | Video | Day 2 |
| 9 | Image segregation into right and left | Video | Day 2 |
| 10 | Image sharing and upload process | Video | Day 2 |

Image capturing: Day 3-11 training at Advanced Eye Centre, day 12-14 at the Primary Health Centre.

**Supplementary Figure 1:** Darkroom conditions at the study sites


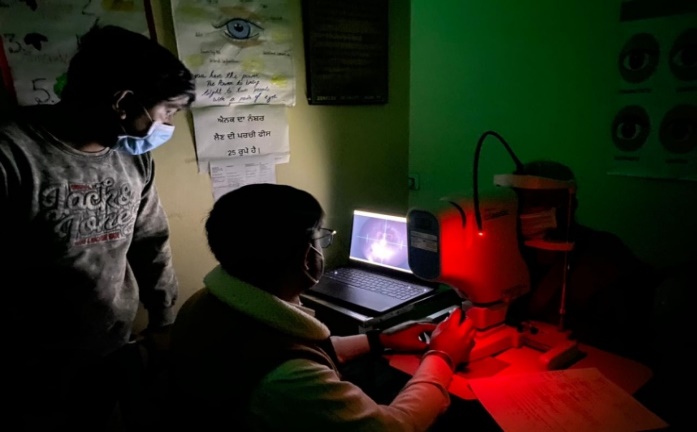

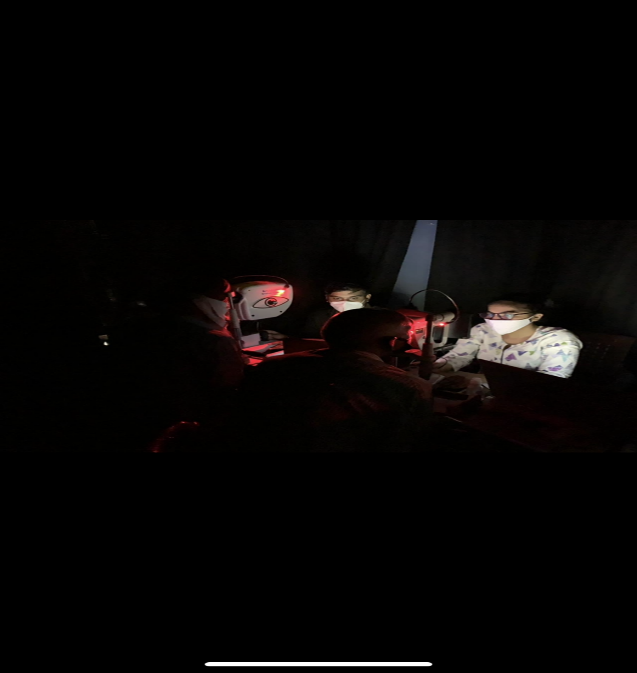


1. Primary Health Centre Khijrabad b) Community Health Centre Badhani Kalan

**Supplementary Figure 2:** Artificial intelligence algorithms screening outputs


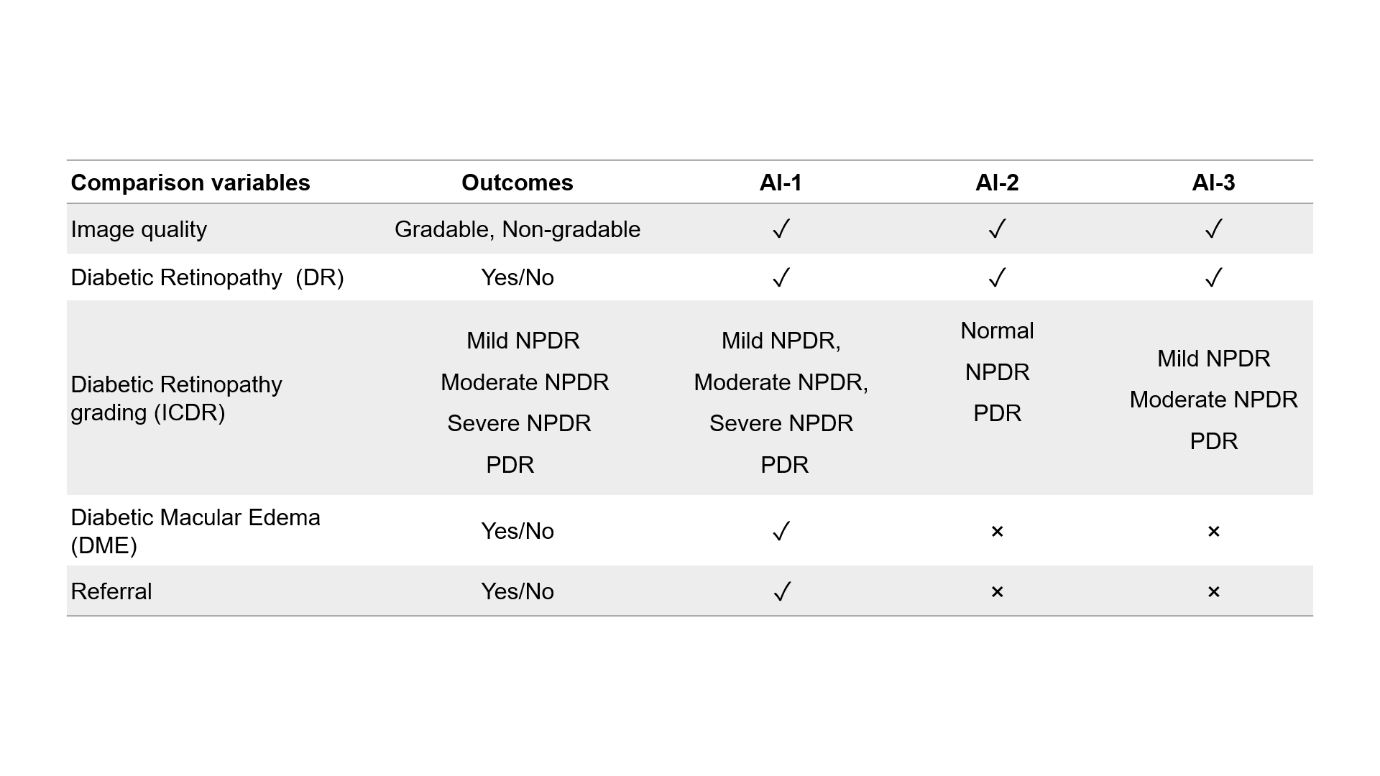


AI - Artificial intelligence, NPDR- Non-Proliferative Diabetic retinopathy, PDR - Proliferative Diabetic retinopathy,

**Supplementary Figure 3:** Integration of AI with the Forus 3Nethra Classic

**Supplementary Table 2**: Diagnostic performance of three Artificial Intelligence algorithms to detect diabetic retinopathy with the Arbitrated Grader Set

| **DR (yes/no)** | **Sensitivity**  **(95% CI)** | **Specificity**  **(95% CI)** | **PPV**  **(95% CI)** | **NPV**  **(95% CI)** | **Accuracy** | **Kappa** |
| --- | --- | --- | --- | --- | --- | --- |
| **HG vs. AI-1** | 97·74  (93·05 – 99·42) | 14·25  (10·85 – 18·45) | 30·16  (25·91 – 34·77) | 94·34  (83·37 – 98·53) | 37·19% | 0·04 |
| **HG vs AI-2** | 59·69  (50·68 – 68·12) | 87·05  (83·15 – 90·63) | 64·71  (55·35 – 73·09) | 85  (80·45 – 88·34) | 79·39% | 0·42 |
| **HG vs AI-3** | 68·42  (59·71 – 76·05) | 96·01  (93·24 – 97·72) | 86·67  (78·31 – 92·26) | 88·92  (85·21 – 91·81) | 88·43% | 0·65 |

DR: Diabetic retinopathy; CI: Confidence interval; PPV: Positive predictive value; NPV: Negative predictive value; AI: Artificial intelligence

**Supplementary Table 3: Suggested changes and incorporation into artificial intelligence**

| **Section** | **Feedback** | **Changes** |
| --- | --- | --- |
| Contact information | Non-editable after initial entry | enabled |
|  | SMS and WhatsApp options are not available for sharing the results | enabled |
| Integration | An Internet connection is mandatory to start image-capturing | not done, internet connection is mandatory |
|  | Loss of images in case of loss of internet connection | cloud backup for intermittent storage |
| Connectivity | Cannot initiate entries without an internet connection | not done, internet connection is mandatory |
|  | Cannot initiate image capturing without an internet connection | not done, internet connection is mandatory |
| Result | No DR stages were provided | added |
|  | DME status not provided | added |
|  | Referral status not provided | added |
|  | Output as Excel/csv file not provided | added |

*DME - Diabetic Macular Edema, DR - Diabetic Retinopathy

Supplementary Figure 4: Receiver Operating Characteristic (ROC) Curves for AI Performance Across Outcome Variables in Diabetic Retinopathy Screening

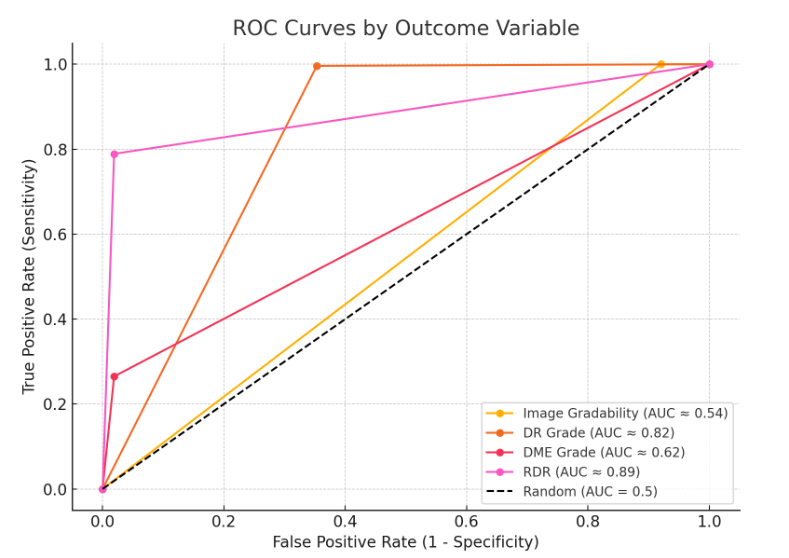


*DR: Diabetic retinopathy, DME: Diabetic macular edema, RDR: Referable diabetic retinopathy

Figure 5: Sample of normal retinal image


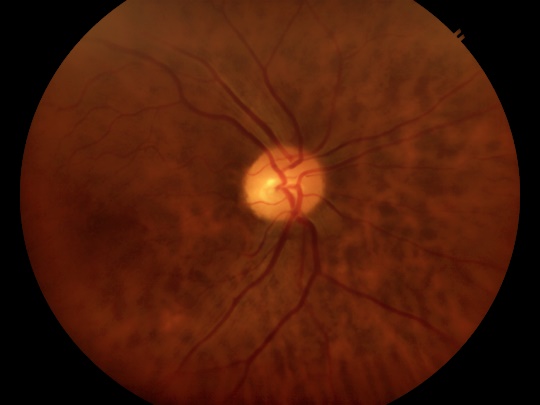


Figure 6: Sample of ungradable retinal image


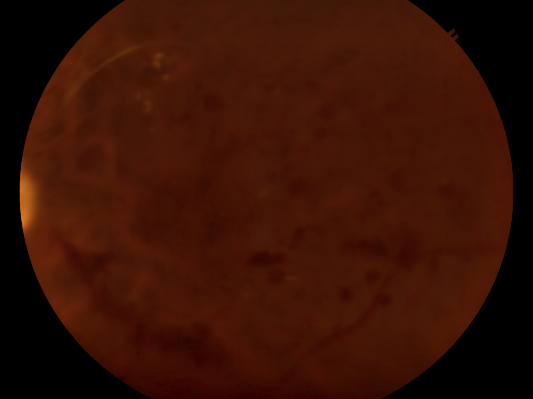

Supplement: Multimedia Appendix 1 [file medinform-v13-e67529-s001.docx]
