# Supplementary material for: Real-World Evaluation of AI-Driven Diabetic Retinopathy Screening in Public Health Settings: Validation and Implementation Study
Source: JMIR Med Inform. 2025 Sep 9;13:e67529. doi: 10.2196/67529 (PMC12419978; doi:10.2196/67529)
Supplement: Checklist 1 [file medinform-v13-e67529-s002.pdf]

iCHECK-DH: Guidelines and Checklist for the Reporting on Digital Health Implementations.

SECTION		ITEM	DESCRIPTION
**TITLE**	1	Title (M[1])	Real-world Validation and Implementation of AI-Based Algorithms for Diabetic Retinopathy Screening in Public Health Settings
**ABSTRACT**	2	Abstract (M)	The abstract has been arranged as Background - Objectives - Methods - Implementation (Results) - Conclusions.<br><br>Background and objectives: explain the need and context of the study<br>Methods: Describe the implementation strategy.
**INTRODUCTION**	3	Context (M)	Added
	4	Problem statement (M)	Added
	5	Similar Interventions (M)	NA
**METHODS**	6	Aims and Objectives (M)	This is the first Indian study to validate multiple commercially available AI algorithms for DRS. It also aimed to assess the technical feasibility of implementation (integration, diagnostic performance) of a validated AI-enabled DRS algorithm in Indian public health settings.<br>**The last paragraph of the introduction explains the aims and objectives**<br><br>The study prospectively validated three DR detection AI algorithms (validation phase) and assessed the technical feasibility of implementing a validated AI algorithm (implementation phase) in public health settings.<br>The first paragraph of the study design explains the study phases linked to the objectives.
	7	Blueprint summary (M)	The study design explains the study intervention:<br>**Study design**<br>The study prospectively validated three DR detection AI algorithms (validation phase) and assessed the technical feasibility of implementing a validated AI algorithm (implementation phase) in public health settings. The STARD checklist (1) was used to report the completeness and transparency of diagnostic accuracy. Figure 1 summarises the overall study design.<br>Available on page 2

[1] M: Mandatory item

8	Technical Design (M)	**The study involved** validating multiple commercially available AI algorithms for DRS and assessing the technical feasibility of implementation (integration, diagnostic performance) of a validated AI-enabled DRS algorithm in Indian public health settings.<br><br>**AI algorithms**<br>Based on a scoping review, five AI-based companies, including four available Indian companies and one international company utilizing cloud-based AI methods for DR detection, were invited. Prior to validation, the AI companies were provided with details regarding the study objectives and the camera and image specifications.<br><br>No AI algorithm was developed during the study, on the other hand the commercially available algorithms were validated and the best performing AI algorithms were integrated into the table top fundus camera and implemented for DRS in a CHC (implementation phase). The details of the algorithms are already published in the study titled:<br><br>M Duggal, A Chauhan, A Kankaria, V Gupta, A Roy, P Verma, et al. Responsible Adoption of Cloud-Based Artificial Intelligence in Health Care: A Validation Case Study of Multiple Artificial Intelligence Algorithms for Diabetic Retinopathy Screening in Public Health Settings. Taylor and Francis 2024 (In press). 2024;
9	Target (M)	The inclusion criteria section on page 4 explains:<br><br>Individuals over 30 years old with a history of diabetes mellitus were screened for DR, per the National Program for the Control of Non-Communicable Diseases (NPNCD), which mandates screening for NCDs, including diabetes, in this age group (2). Informed consent was obtained, and no changes were made to their routine care during recruitment.
10	Data (M)	The fundus imaging data management has been explained in the fundus image acquisition section under the subsection:<br><br>Fundus image grading<br>The image identifiers were removed, and the images remained unprocessed before AI analysis. The research optometrist sorted images into left-eye and right-eye folders and uploaded them for AI output. An agreement was established with the AI companies to ensure that only the uploading optometrist had access to fundus images, with the AI companies having no direct access to the images. A separate account was created for image upload and grading, with access restricted to the optometrist through role-based access controls. Audit logs tracked the access to the images, preventing unauthorized access, and secure transmission file transfer protocols were used to protect the images during transfer. Similar access was provided to the individual human grader for image grading. The

		images captured during the validation phase were not used for AI training or testing.<br>Available on page 4.<br><br>The patient's consent has been explained in the inclusion and exclusion criteria.
11	Interoperability (M)	No new tool or systems was developed during the study
12	Participating entities (M)	**Implementing organization:** The Postgraduate Institute of Medical Education and Research (PGIMER) is a premier tertiary care institution in India, designated as an Institute of National Importance. Renowned for its excellence in medical education, cutting-edge research, and high-quality patient care, PGIMER operates under a clear mission to develop patterns of postgraduate medical education in all its branches, aiming to produce competent specialists committed to the highest standards of healthcare. Its leadership and vision are grounded in advancing healthcare delivery, fostering innovation, and addressing national and regional health priorities.<br>Mission, leadership, vision, etc. can be found in more detail in https://pgimer.edu.in/PGIMER_PORTAL/PGIMERPORTAL/home.jsp<br><br>**Government involvement and role:** The Punjab Health Department, Civil Surgeons of Mohali and Moga, Senior Medical Officers of Boothgarh and Badhani Kalan, and the Deputy Commissioner of Moga implemented the study.<br>During the implementation phase, two optometrists from the Punjab health system in Moga district were nominated and received fundus image acquisition training.<br>The integration phase focused on ensuring seamless hardware and software compatibility, assessing internet connectivity, and conducting dummy tests to validate preliminary outputs before implementation. It was carried out in district hospital Mohali with the support of the Punjab health administration.<br><br>**Funders:** The study was funded by the National Institution for Transforming India (NITI) Aayog, the Government of India.<br><br>No new product requiring intellectual property rights was developed after the implementation phase.
[redacted]		
13	Budget Planning (M)	The budget was allocated across designated heads—salary, equipment, recurring expenses, travel, and contingencies—for a project duration of 14 months. Per the funding agency's guidelines, actual expenditure details are not permitted to be disclosed publicly by the funding agency.

	14	Sustainability (M)	During implementation, capacity was built within the public health system by training optometrists and non-specialist staff to perform AI-based retinal imaging. Post-study, a follow-up mechanism was established to ensure continued service delivery (6 months), with the optometrist monitored for adherence to screening protocols. Notably, the retinal camera and adjustable stand were retained at the Community Health Centre (CHC) rather than being reclaimed as study assets, reinforcing sustainability through local ownership and continuity of service. This has been added in the AI integration and implementation paragraph on page 6.
**RESULTS**	15	Coverage (M)	The study's validation and implementation phases were conducted in the Mohali and Moga districts of Punjab, India. 250 participants were enrolled during the validation phase, while 343 individuals were recruited for the implementation phase.
	16	Outcomes (M)	**AI integration and implementation**<br>Technical feasibility: The better-performing AI algorithm from the validation phase was integrated into the 3Netra Classic and pilot-tested for two weeks at AEC and District Hospital Mohali. The integration phase focused on ensuring seamless hardware and software compatibility, assessing internet connectivity, and conducting dummy tests to validate preliminary outputs before implementation.<br>Available of page 5<br><br>**Implementation Phase :**<br>AI-assisted DRS was deployed at Community Health Centres (CHCs) during the implementation phase. This AI was assessed against the reference standard for evaluating AI performance and was used for subsequent statistical analysis of diagnostic accuracy.<br>Page 5
[redacted]	17	Lessons learned (M)	Yes, several operational challenges were encountered during the study's implementation phase. These are detailed in a separate manuscript titled "Operational Challenges and Adaptive Strategies for Diabetic Retinopathy Screening in Public Healthcare Centres in India," which is currently under review. The paper presents an in-depth analysis of key barriers, including limited patient accessibility, infrastructural constraints, ergonomic limitations, suboptimal imaging conditions, and the adaptive strategies employed to address them.
	18	Unintended consequences (NM[2])	NA
**DISCUSSION**	19	Conclusion (M)	In conclusion, this study highlights the essential role of systematic AI validation in integrating technology responsibly into clinical workflows. By demonstrating AI's feasibility for DRS in Indian public health settings, our findings support scalable solutions to improve healthcare accessibility in resource-constrained contexts across the Global South. However, long-term sustainability and large-scale implementation will require continued funding, workforce capacity,

[2] NM : Non-mandatory item

			and policy integration. Further research is needed to evaluate large-scale deployment, examining clinical effectiveness, cost-effectiveness, and the challenges of implementing AI-driven screening strategies in real-world settings.<br><br>Page 11
**GENERAL**	20	General (NM)	NA

References

1. Cohen JF, Korevaar DA, Altman DG, Bruns DE, Gatsonis CA, Hooft L, et al. STARD 2015 guidelines for reporting diagnostic accuracy studies: explanation and elaboration. Available from: http://dx.doi.org/10.1136/bmjopen-2016-012799
2. Directorate General of Health Services Ministry of Health and Family Welfare Government of India. National program for prevention and control of cancer, diabetes, cardiovascular diseases and stroke (NPCDCS) Directorate General of Health Services Ministry of Health and Family Welfare Government of India Handbook for counselors reducing risk factors for noncommunicable diseases risk factors for NCDs [Internet]. [cited 2023 Oct 11]. Available from: https://main.mohfw.gov.in/sites/default/files/Handbook%20for%20Counselors%20-%20Reducing%20Risk%20Factors%20for%20NCDs_1.pdf
